# Socio-Economic Status and Bullying Victimization in India: A Study About Social Misfit and Minority Perception

**DOI:** 10.1007/s10964-024-02021-7

**Published:** 2024-05-29

**Authors:** Niharika Thakkar, Mitch van Geel, Maike Malda, Ralph Rippe, Paul Vedder

**Affiliations:** 1 Department of Child and Adolescent Studies, Leiden University, Leiden, the Netherlands; 2 Downsideup Academic Coaching, Randstad, the Netherlands

**Keywords:** Bullying victimization, SES context, Minority perceptions, India, Growth modeling

## Abstract

The Social Misfit Theory, which states that some individuals deviate from what is normative in a community and may therefore be more likely to be victimized, has mostly been studied in Western countries. The current study addresses in a longitudinal sample whether socio-economic minorities (SES) in the classroom (a contextual SES minority) are more likely to become victims of bullying in India, and whether the relation between minority status and victimization is mediated by perception of oneself as a minority. The current study used three waves separated by three month intervals. A sample of youth from Indore India (grades 7 to 9; *N* = 1238; *M*-age_T1_ = 13.15, *SD* = 1.16, 24 percent girls) was used. It was found that being a contextual SES minority was related to more victimization, but only when the contextual status was corroborated by the perceived minority status. However, over time, being part of a contextual minority predicted decreased victimization, possibly pointing to normative beliefs and values in the Indian context. The results of this study are in contrast to the Social Misfit Theory, but do support self-perception as a mediator.

## Introduction

The prevalence of bullying and victimization among school going adolescents has been recognized and documented globally (Elgar et al., 2015). Bullying is a subtype of aggressive behavior, in which an individual or a group of individuals repeatedly attacks, humiliates, and/or excludes a relatively powerless person (Salmivalli, 2010, p. 112). Though some proportion of youth in any culture and community will perpetrate or experience bullying victimization irrespective of background (Durkin et al., 2012), some youth may be more likely to experience victimization than others. One theory that attempts to explain why some youth are more likely to be victimized is the Social Misfit Theory (Wright et al., 1986). This theory states that some individuals deviate from what is normative in a community or, as in the current study, in a classroom, and may therefore be more likely to be victimized. However, almost all work on the Social Misfit Theory and victimization was done in the Western World, and no study addressed the Social Misfit Theory as an explanation for bullying in India. To address this gap, the current longitudinal study aims to investigate cross-sectionally, as well as over-time, whether Social Misfit is related to bullying among youth in India, using both self- and peer-reports of bullying and victimization. In the current study the focus is on Social Misfit with regards to socio-economic status (SES), a variable that may be particularly important to youth in India.

### Bullying and Social Misfit

Several studies, almost exclusively from the Western world, show support for the Social Misfit Theory with regards to bullying victimization. Indeed, interviews with adolescents reveal that victims of bullying are seen by adolescents as ‘different’ or ‘wrong’ (Thornberg & Delby, 2019). Some of the support for the Social Misfit Theory in the classroom comes from studies about ethnic diversity; ethnic Dutch youth reported more victimization experiences with fewer ethnic Dutch children in the classroom, whereas Turkish, Moroccan, and Surinamese minority youth reported more victimization in classroom with more ethnic Dutch children in the classroom (Verkuyten & Thijs, 2002). In a study from Sweden, immigrant youth were found to be more likely isolated in immigrant sparse classrooms, whereas majority youth were more likely to be victimized in immigrant dense classrooms (Plenty & Jonsson, 2017). However, support for the Social Misfit Theory is not limited to ethnicity in the classroom. A recent longitudinal study (Kaufman et al., 2022) extends the Social Misfit Theory beyond ethnicity and shows that adolescents who deviate from the classroom norm in terms of friendship, social media connections, social anxiety, and disruptive behavior were more likely to be victimized over time. Dissimilarity between individual and classroom in terms of personality has also been found related to higher self-reported peer victimization (Boele et al., 2017). Taken together, those who differ from the group norms, be it in terms of behavior, ethnicity, or personality, may be more likely targets for bullying victimization, and it has even been suggested that bullying is a method to force deviant individuals to adhere to the group norms (Juvonen and Galván, 2009).

### Bullying and SES

Past studies have found that SES plays a small but significant role in bullying and victimization among adolescents. Tippett and Wolke’s meta-analysis (2014) suggests that children from lower SES households experience harsher punishment, restrictive and authoritarian parenting practices, greater levels of sibling violence, and are more often exposed to incidents of domestic violence. Through observational learning, vicarious experiences, and modeling (Bandura, 1978), these experiences of violence or abuse at home may shape children’s interaction with peers, adversely affecting their ability to form or maintain peer relationships. This predisposes lower SES children rather than higher SES children to higher risk for victimization through indirect factors instead of directly observed socio-economic levels (Due et al., 2009; Tippett & Wolke, 2014). Past studies from India, albeit very few, have also found that SES contributes to distinguishing students who were involved in bullying from those who were not (Sethi et al., 2019). Malhi et al. (2015) found that low SES students scored higher on physical victimization, whereas high SES students scored higher on relational victimization.

### Research in India

India houses the largest adolescent population in the world, 356 million youth between the ages of 10 to 19 years (United Nations Population Fund, 2014), harboring an enormous repository of adolescent behavior carrying vast potential to contribute to social science research. Unfortunately, especially compared to the west, few scientific articles exist that address bullying among youth in India (Thakkar et al., 2020a). Indian society is hierarchical, and marked by disparities in socio-cultural factors such as SES, religion, caste, gender, and color. For example, adolescents belonging to groups with lower SES in India often have responsibilities at home that involve tasks like cooking, cleaning, or fetching water from the nearest water source because continuous water supply is not readily available to low-income households (Bapat, 2016). Similarly, access to material resources such as electricity, sanitation facilities, cars, internet, and technology varies unequally among children based on their family’s SES. Consequently, there are differences in the daily lives of young people due to SES inequality. Some children shoulder responsibilities of contributing to the household income by taking up paid labor jobs, while more economically privileged children focus on academic achievements (UNICEF, 2019). These circumstances impact school attendance, academic performance, and interpersonal relationships among students within the classroom. The availability or lack of material wealth and resources serve as indicators of the youth’s context in India, influencing their experiences either positively or negatively, and contributing to group dynamics that may affect bullying and victimization in classrooms. Furthermore, the normative acceptance of the abovementioned status hierarchies, cultural disparities, and socio-economic inequalities, typical of India (Panda & Gupta, 2004), leads to a segregated society which reflects a power imbalance (Campbell et al., 2018). Together these notions lead us to suggest that being Social Misfits or being at the lower end of a power imbalance in a community as regards SES is related to bullying victimization in India. The focus in the current study is on the classroom as opposed to the whole school because most theory about bullying in youth, as well as existing instruments, focus on the classroom level as opposed to the school level (see also Salmivalli, 2010), and thus a focus on the classroom ensures connections with previous research and avoids a potential bias in our instruments. There are few studies about bullying in India (Thakkar et al., 2020a); in order to get a better grip on context, a study in India therefore had to be similar with regards to other variables, and therefore we choose to include an age group that is often the focused in existing literature about predictors of victimization (see for example Kljakovic & Hunt, 2016)

### Contextual SES and Perception of Contextual Status

In line with the Social Misfit theory, a contextual minority is in this study defined as a minority with regards to SES in their classroom; for example a low SES student in a classroom consisting mostly of high SES classmates, or a high SES student in a classroom that consist mostly of low SES classmates. The effect of being a Social Misfit on being victimized may be either direct or indirect. Direct means that peers perceive of a student as a member of a minority in the classroom and start negative interactions with or around the minority student. Indirect refers in the current study to the mediation of bullying and eventual victimization by the victims’ self-perception. During classroom interactions, the process in which individuals appraise or view themselves, hence the perception of their contextual status contributes to their adjustment in the classroom (Verkuyten & De Wolf, 2002). According to the self-categorization theory (Turner, 1987), people perceive themselves to be members of various groups. They categorize themselves as either a member of a group or as a non-member. Categorizing as a member results in increased conformity to in-group stereotypes and a maximization of differences with out-group characteristics (Verkuyten & De Wolf, 2002). Hutnik (2004) even suggested that *perceptions* of group membership, e.g., seeing oneself as member of a lower SES group, may be relatively more important for explaining victimization than either personal SES or contextual SES. Personal SES is defined by cultural or material resources available to students in their homes and contextual SES refers to how the personal SES compares to the personal SES of classmates. Such comparisons can be conducted by individual students and then result in their *perceived* contextual SES, e.g., as either belonging, or not, to the majority or the minority group in the classroom. In high power-distance countries like India, with a wide and uneven distribution of authority, resources, and privileges (Hofstede, 2011), the sense of powerlessness perceived by “out-group” or minority individuals may promote a fatalistic attitude of apathy and hopelessness, that could lead to an acceptance of bullying behaviors (Verma, 2004).

## Current Study

Though several studies about the Social Misfit Theory and bullying have been conducted, none of these studies were done in India. The current study aims to provide a report on Social Misfit with regard to SES and bullying victimization in India, particularly focusing on SES mismatch within a context rather than an individual’s objective SES, using self- as well as peer-reports to measure victimization. It is expected that SES *contextual minorities* experience higher levels of victimization than *contextual majorities* at baseline T1, T2 and T3, and also longitudinally over time from T1 to T2 to T3 (Hypothesis 1). Specifically, in a classroom of low SES majority, middle and high SES students experience higher levels of victimization than low SES students. In a classroom of high SES majority, low and middle SES students experience higher levels of victimization than high SES students, and in a classroom of middle-income majority, low and high SES students experience higher levels of victimization than middle SES students. Furthermore it is expected that the associations between *contextual minorities* and victimization are mediated by individuals’ *minority perceptions* at baseline and over time, such that, *contextual minorities* who perceive themselves as minorities, subsequently experience higher levels of victimization as compared to *contextual majorities* (Hypothesis 2).

## Methods

### Participants

Data were collected from nine schools in and around the city of Indore in central India at three time-points with intervals of three months in the school year of 2015–2016. A total of 1238 students (grades 7 to 9; aged 11–16 years, *M*_age_ = 13.01, *SD* = 1.15) were included in the analyses (1120 at T1- 296 girls, 824 boys; 1,036 at T2- 274 girls, 762 boys; and 1006 at T3- 282 girls, 724 boys). Students completed the questionnaire in either Hindi (*N* = 497; 40%), India’s national language, or English (*N* = 741; 60%), depending on the formal language of instruction of the participating schools. Of the nine participating schools, three were public schools (i.e., funded and run by the government) whereas six were private schools (privately owned by non-government organizations). In all schools, students were with the same teacher the whole day. Eight schools were co-ed schools, which means mixed boys and girls’ schools, whereas one school was an all-boys school. Large class sizes with sometimes over 50 students sitting closely together, combined with laxed disciplinary structures in classrooms have long been identified to complicate data collection processes in India (Bapat, 2016). The current study is also affected by this, and, therefore, some exclusions in data were made to eventually maintain a sample that is consistent with global research standards. The initial sample consisted of 1908 students from ten schools, between the ages of 11 to 16 years, from grade 7, 8, and 9. From the all-boys school 143 students at T2 were excluded from data collection, due to disturbances and laxed discipline in classrooms. From Grade 7 of one school, 185 students had received two sets of questionnaires during data collection at T1, one in English and the second in Hindi the next day, because the students found the English questionnaires difficult to follow on day 1 despite the medium of instruction for that school being English, thus, excluding these students from final analyses. One of the ten participating schools chose to drop out in Wave 3 because of undisclosed reasons and thus all students (337) from that school were excluded from the analyses. Five students were excluded due to incomplete data on their grade. Consequently, the final sample consisted of 1238 students from nine schools. A total of 43 classrooms were included in the final sample, with an average grade strength of *M* = 33.5 (*S.D* = 9.2). Descriptive statistics for age, SES, and victimization scores of the participants are reported in Table 1. Beyond the abovementioned exclusions, students that opted out of the research or were absent during data collection (118 at T1; 202 at T2; and 232 at T3) were marked as missing in analyses. A record telling the absentees apart from the students who opted out was not maintained. All attention focused on the students filling out the questionnaires by addressing their questions and keeping them at task during data collection. Several analyses were performed to compare the excluded students (*N* = 724) with the final sample. Independent sample t-tests showed that at T1, there was no significant difference on the variables of age, religion, caste, and mean scores on the self-reported victim scale, however, there was a significant difference between the two groups in the percentage of times a child was peer-reported as a victim (*F* (1951, 1946.98) = 79.31, *p* < 0.001) and on sum scores for the Family Affluence Scale (*F* (1698, 1289.11) = 9.08, *p* < 0.001). Furthermore, students that were present at all three waves of the study (*N* = 795) were compared to students that were present at point T1, but absent at T2 and T3 (*N* = 63), or students that were present at T1, but absent at either T2 (*N* = 113) or T3 (*N* = 149). Independent *t*-test analyses showed that the two groups were not significantly different on SES, but were significantly different on age at T1 (*F* (1123, 591.37) = 2.58, *p* < 0.05) such that the students who were present at all three waves were significantly younger than students who were absent at either T2 or T3, or both. Chi-square tests revealed that the two groups did not differ on caste and religion but the proportion of males present at all three waves was significantly higher (χ^2^ (1) = 12.77, *p* < 0.001) as compared to the absentee group.

**Table 1:** Descriptive statistics and zero-order correlations of variables in the study

	1	2	3	4	5	6	7	8	9	10	11	12	13
1. Age (T1)													
2. Age (T2)	0.85**												
3. Age (T3)	0.83**	0.85**											
4. Individual SES; T1	−0.12**	−0.10**	−0.10**										
5. Individual SES; T2	−0.16**	−0.14**	−0.13**	0.75**									
6. Individual SES; T3	−0.17**	−0.15**	−0.14**	0.76**	0.81**								
7. Self-report victim (T1)	−0.04	−0.05	−0.04	0.00	−0.05	0.00							
8. Self-report victim (T2)	0.03	0.01	0.01	0.06	0.02	0.03	0.52**						
9. Self-report victim (T3)	0.01	0.01	0.01	0.06	0.05	0.02	0.42**	0.49**					
10. Peer-report victim (T1)	−0.06	−0.04	−0.06	0.12**	0.14**	0.12**	0.12**	0.10**	0.09**				
11. Peer-report victim (T2)	−0.02	0.03	−0.05	0.11**	0.11**	0.11**	0.22**	0.19**	0.12**	0.48**			
12. Peer-report victim (T3)	−0.10**	−0.08*	−0.14**	−0.03	0.02	−0.01	0.13**	0.14**	0.10**	0.42**	0.38**		
13. Minority Perception	0.01	0.04	0.03	0.21**	0.19**	0.15**	0.07*	0.08*	0.02	0.11**	0.13*	−0.02	
* n*	1125	1028	1014	1118	1027	995	1084	1014	987	1233	1235	1236	1082
* M*	13.15	13.32	13.60	4.91	5.11	5.17	2.13	2.16	2.18	16.49	28.89	26.72	2.59
* SD*	1.16	1.21	1.18	2.29	2.29	2.25	1.10	1.13	1.13	13.97	19.11	15.93	1.19
Range	10	8	7	9	9	9	4	4	4	94	80	89	4

T1 = Time Point 1, T2 = Time Point 2, T3 = Time Point 3

*p < 0.05; **p < 0.01

### Procedure

The Institutional Review Board of the Institute of Education and Child Studies at Leiden University approved of the study. A convenience sample was obtained by approaching 15 schools in the school year 2015–2016. Ten schools agreed to participate. No compensation was offered to any schools at the outset, however, four of the participating schools requested it in conversation with the researchers, of which three schools were given vouchers to a bookstore for each wave, whereas one school was given carpets for the students to sit on in the classroom. No student was offered independent compensation for their participation. Instructions to students included that their participation was voluntary, and would bear no consequence on their academic performance, or have any other implications, neither positive nor negative. Students were also informed that their information/responses would be kept confidential and not shared with parents, teachers, or classmates. At the discretion and recommendation of the principals of the participating schools, the principals, substituting as responsible consenting adults for the students in a school setting (Malamut et al., 2020), gave written consent to collect data from students in grades 7, 8, and 9. Principals were informed of all the features of the research that could affect their willingness to allow the child to participate. Students were allowed to opt out of the research. Every student enrolled in a class at the time of data collection was invited to complete the questionnaire, and while most students chose to participate, some students chose to go the library or complete their home work in the back rows of the class. Students who thus opted out of research were marked as absent (missing) in analyses. The questionnaires were distributed to the students in their classrooms during a pre-arranged time. There was a team of 20 trained research assistants, who were all first- or second-year master students of Social Work. During simultaneous data collection in multiple grades, at least two research assistants were present in each class, gave instructions and were available to answer any of the students’ questions. Class teachers helped to keep students on task but were asked not to interfere with completing the questionnaires. The students took approximately 75 min for each round to complete the full questionnaire.

### Measures

Students provided information regarding socio-demographics like gender, grade, age, religion, caste, languages spoken, and family composition and affluence through self-reported questionnaires. The original English scales used in the present study were translated to Hindi through a formalized translation procedure following guidelines laid by Beaton et al. (2000). A more detailed account of the translation procedure can be found elsewhere (Thakkar et al., 2021).

#### Self-reported SES

The Family Affluence Scale II (FAS; Currie et al., 1997) was used at T1, T2, and T3 to measure individual socio-economic status, and used as input to calculate contextual minorities and majorities; we explain the calculation in the analysis subsection. This self-report measure consists of four questions, each using a different response scale. The four questions in the scale are, “Does your family own a car, van, or truck?”, “Do you have your own bedroom for yourself?”, “During the past 12 months, how many times did you travel out of town on holiday with your family?”, and “How many computers or laptops do your family own?”. The FAS was developed so that adolescents can give an approximation of their SES. The FAS has been found to be a valid indicator of SES (Boyce et al., 2006), and has been validated for its use with Indian adolescents (Bapat, 2016). Test-retest correlations between Wave 1 and Wave 2, Wave 2 and Wave 3, and Wave 1 and Wave 3 were found to be *r* = 0.73, *r* = 0.79, and *r* = 0.75 for the English questionnaires, and *r* = 0.70, *r* = 0.77, and *r* = 0.65 for the Hindi questionnaires.

#### Self-reported bullying victimization

The victimization subscale of the Illinois Bully-Fight-Victim Scale (Espelage & Holt, 2001) was used at T1, T2, and T3, to assess self-reported bullying victimization. The scale has been found valid and reliable (Espelage et al., 2003). The victimization scale consists of four items that measure the experience of victimization from peers. The four questions of the scale are “Other students made fun of me”, “Other students called me names”, “I got hit and pushed by other students”, and “Other students teased me”. Response options for the scales are *never* (1), *1 or 2 times* (2), *3 or 4 times* (3), *5 or 6 time*s (4), and *7 or more times* (5) in the past 30 days. In the present study, Cronbach’s alpha for this scale was found to be 0.81 at T1, 0.84 at T2, and 0.85 at T3 for the English questionnaires and 0.88 at T1, 0.90 at T2, and 0.92 at T3 for the Hindi questionnaires.

#### Peer-reported bullying victimization

All students in the classroom were given a sheet of paper that described bullying behavior on the top in a few words (repeatedly teasing, fighting, excluding, name-calling, etc). Self-reports are frequently used in the study of bullying behaviors and provide information with regard to personal experiences, but also run the risk of being biased due to shared method variance (Cornell & Bandyopadhyay, 2009). On the other hand, peer-reports provide a report through multiple informants and decrease measurement error (Branson & Cornell, 2009). For reliable results and to assure the validity of the constructs measured, past researchers advise the use of a combination of both type reports in the study of bullying and victimization behaviors (Van Geel et al., 2018).

Students were asked to nominate (circle names of) victims of bullying from a list of their classmates at each of the time-points T1, T2, and T3. The number of victims to be listed was not limited. Dyadic nominations of bully and victim status, received by peers from within a classroom, are found to be a reliable and valid estimate yielding consistent results with other informant reports across studies, (Malamut et al., 2020) as well as in the Indian setting (Thakkar et al., 2021). A total score was computed based on the number of times an individual was marked as a bullying victim by their classmates. This total score was changed into proportions by dividing the total score by the number of students in class, as suggested and done in earlier studies (Veenstra et al., 2007).

#### Perception of minority or majority status

The authors of the study designed a questionnaire to measure if individuals perceived themselves as a minority or majority at T1 in their classroom on family income, referred to as *minority/majority perceptions* in this paper. Self-reported indicators of family income have been found to be valid measures of socioeconomic index (Tippett & Wolke, 2014). Students were asked to respond to the question “How many classmates have the same financial condition (family income) as your family does?” on a five-point scale ranging from “*none*”, *“some*”, “*about half*”, “*many*”, and “*all*”, where a lower score is indicative of a perception of minority, and higher score is indicative of perception of majority in a continuous capacity.

### Analysis Plan

In the present study, we used a growth model framework to incorporate a mediated moderator approach in longitudinal capacity, with full information maximum likelihood (FIML; Schlomer et al., 2010) estimation to allow for missing values, to study the effects of contextual *minority SES* and *majority SES* status and *minority/majority perceptions* of the status (mediator), on victimization within classroom in Indian school-going youth. All main analyses were conducted in *R version 4.0.2* (R Core Team, 2020).

#### Statistical power

The large sample size of more than 1000 participants, having over 10 classes, 2 main groups of self- and peer-reported victimization scores, and 3 timepoints, 5% significance level and high power of 0.95, according to a repeated measures design G*power, enabled us to detect a (very) small group by time interaction effect size of *f* = 0.046. This suggests that the current sample provides sufficient power to detect any effect size (f) exceeding 0.046.

#### Victimization

For the self-reported victim scale, we computed means for students who had responded to 80% or more items on the total four questions of the Illinois victim scale for T1, T2, and T3 respectively. The 80% cut-off rule was implemented as it is the criterion proposed by the authors of the scale (Espelage & Holt, 2001). Students who had incomplete data on more than 20% items on the scale in a particular wave were defined as missing for the total score. These missings were handled using a Full Information Maximum Likelihood (FIML) estimation in the main growth model. For the peer-reported victim scales, percentage of times a child was marked a victim in class was calculated by classroom size (count*100/total number of students in class) (Veenstra et al., 2007).

#### Socio-economic status

To estimate a student’s SES status, at step 1 a mean score for students on each of the four FAS items from each wave was calculated. For example, *Mean* FAS item 1 = (FAS_T1_ + FAS_T2_ + FAS_T3_) / 3. Similarly, *Mean* scores for FAS items 2, 3 and 4 were calculated for each student. Reliability analysis to check for stability of SES scores across waves confirmed that reliability (Cronbach’s alpha) between item level scores for individual FAS items was 0.86, whereas Cronbach’s alpha for sum scores of FAS at T1, T2, and T3 was 0.92. Given this consistency in SES scores across waves, it was deemed feasible to calculate a mean score for SES items, thereby also deriving a more durable SES estimate for each student. At step 2, a composite SES score was calculated for each student based on their *Mean* scores on four FAS items (Currie et al., 1997). We used a three-point ordinal scale, where SES low (score = 0,1,2) indicates low affluence, SES medium (score = 3,4,5) indicates middle affluence, and SES high (score = 6,7,8,9) indicates high affluence (Boyce et al., 2006). These cut-off scores have been validated in a study with an Indian sample, and were found to be reliable (Bapat, 2016). In the present study, 16.1% (*n* = 180) students qualified as low SES, 42.8% (*n* = 478) students qualified at middle SES, and 41.1.% (*n* = 460) students qualified as high SES in Wave 1. In Wave 2, 13.3% (*n* = 137) students qualified as low SES, 42.4% (*n* = 435) students qualified at middle SES, and 44.3.% (*n* = 455) students qualified as high SES. In Wave 3, 12.6% (*n* = 125) students qualified as low SES, 41.5% (*n* = 413) students qualified at middle SES, and 45.9% (*n* = 457) students qualified as high SES.

#### Contextual minorities and contextual majorities

For classroom SES composition, each classroom is distributed into the 3 SES proportions, i.e., percentage of students that classify as low SES, middle SES, and high SES, based on the SES classes as defined above. The class that had the highest percentage of students in each classroom was labeled as “*contextual majority*”, whereas the other two classes were then “*contextual minorities*”. A 5% minimum difference in proportional size criterion was set to allow for well-separated identification of a true minority group in a classroom. For example, without the 5% minimum difference rule, if a particular class had 33% students classifying as low SES, 33% as middle SES, and 34% as high SES, the high-income group could be strictly taken as a majority, whereas both low- and middle-income students would classify as minorities, however, this could be a draw distribution. These draw distributions were neither classified as minority nor as majority. Therefore, a distribution with a minimum 5% difference in proportions, for instance where 38% students classify as high income, 31% as middle, and 31% as low income, was followed to establish unbiased estimates. Given the focus of the present study on SES mismatch beyond individual SES, only those students who qualified as either minority or majority in the context of the classroom, whether high, middle, or low SES, were included in the growth curve model moderator analyses. Applying the clarified classification rule, we found that approximately 12% (*n* = 142) of the students qualified as minorities whereas 65% (*n* = 782) of the students qualified as majorities in the present study.

#### Growth models

**Fig. 1:**
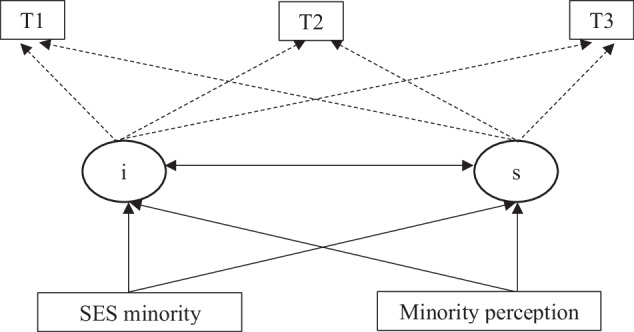
Growth model for baseline victimization and change in victimization over time predicted by minority status mediated through minority perception. *Note.* i = victimization at baseline at T1, T2, and T3; s = victimization change over time from T1 to T2 to T3.

In the growth model, the individual perception of family SES as compared to other students’ family SES was added to examine if the effect of perception of a students’ SES status as minority or majority explained the relationship between *contextual SES* and victimization. For main analyses, a set of five linear growth models with robust standard errors were fitted to evaluate individual as well as classroom level effects, on victimization development over time through the mediator (see Fig. 1). Each of these models were run separately for self-reported victimization and peer-reported victimization to examine the differences and consistency between the self- and informant approach in bullying victimization behaviors (Cornell & Bandyopadhyay, 2009). In Fig. 1, the intercept (*i*) represents victimization (individual baseline differences) as a latent variable at T1, T2, and T3, whereas the slope (*s*) represents the change in victimization over time from T1 to T2 to T3. Model I and II are first examined to test hypothesis 1. Model I (M1) refers to a linear growth model, where both *i* and *s* are predicted by SES *contextual minority* status. Model II (M2) refers to linear growth where *minority perception* is added to the model, and both *i* and *s* are predicted by both *minority status and minority perception*.

For the mediation analyses addressing hypothesis 2, the 4-step causal effect approach as proposed by Baron and Kenny (1986) was incorporated into each of the growth models III, IV, and V as detailed below. It was examined if change in victimization over time is predicted by *contextual minority* status, when baseline differences of victimization at T1, T2, and T3 are predicted by *contextual minority* status and *minority perception*, and whether victimization at *baseline* (M3) is mediated by *minority perception*, or whether *change* in victimization over time is mediated by *minority perception* (M4).

Finally, in model V (M5) it was examined if *change* in victimization over time is predicted by *contextual minority* status and *minority perception*, when baseline victimization is predicted by *contextual minority* status and *minority perception* with mediation of baseline differences in victimization through *minority perception*. Thus, In the above models, all model parameters and standard errors are estimated using robust estimators for skewness.

Descriptive statistics for main variables in the study are reported in Table 1. In the present study, missing value analysis indicated that data were missing completely at random (MCAR; Little, 1988). FIML estimation is a sophisticated procedure known to adequately deal with missing data even they are not MCAR, and, thus, all statistics reported in the analyses used the FIML estimation (Schlomer et al., 2010). The intraclass correlations for the victimization variables, for both self- and peer-reported measures at T1, T2, and T3 were found to be in the range of 0.02 to 0.30 which is considered to be negligible (Shieh, 2016), thus not requiring formal multilevel modeling to account for school or higher order nesting. The potential residual effects of higher order nesting of variance components for the natural variability of the main effects were addressed through robust standard error estimation (Tabatabai et al., 2014). A summary of all growth models is provided in Table 2.

**Table 2:** Growth model summary for self- and peer-reported victimization

	Independent Variable	Dependent Variable	Estimate	SE	*z*
	Self-reported Victimization				
Model 1	Minority status	Victimization at baseline	0.14	0.09	1.58
	Minority status	Victimization change	−0.10	0.05	−1.94
Model 2	Minority status	Victimization at baseline (joint predictors on the left)	0.14	0.09	1.57
	Minority perception		0.07	0.03	2.80**
	Minority status	Victimization change (joint predictors on the left)	−0.11	0.05	−2.03*
	Minority perception		−0.2	0.02	−1.25
Model 3	Minority status	Victimization at baseline	0.13	0.09	1.53
	Minority status	Minority perception	2.36	0.58	4.06**
	Minority perception	Victimization at baseline	0.05	0.02	2.58**
	Minority status	Victimization change	0.11	0.05	−1.99*
Model 4	Minority status	Victimization change	−0.10	0.05	−1.92
	Minority status	Minority perception	2.36	0.59	4.04**
	Minority perception	Victimization change	0.01	0.01	0.73
	Minority status	Victimization at baseline	0.13	0.09	1.48
Model 5	Minority status	Victimization at baseline	0.14	0.09	1.57
	Minority status	Minority perception	2.35	0.58	4.07**
	Minority perception	Victimization at baseline	0.07	0.03	2.80**
	Minority status	Victimization change (joint predictors on the left)	−0.11	0.05	−2.03*
	Minority perception		−0.02	0.02	−1.25
	Peer-reported Victimization				
Model 1	Minority status	Victimization at baseline	−5.25	1.68	−3.14**
	Minority status	Victimization change	1.24	0.72	1.72
Model 2	Minority status	Victimization at baseline (joint predictors on the left)	−5.40	1.77	−3.06**
	Minority perception		1.29	0.31	4.17**
	Minority status	Victimization change (joint predictors on the left)	1.27	0.78	1.64
	Minority perception		−0.47	0.16	−2.95**
Model 3	Minority status	Victimization at baseline	−5.40	1.75	−3.08**
	Minority status	Minority perception	2.33	0.59	3.93**
	Minority perception	Victimization at baseline	0.99	0.28	3.60**
	Minority status	Victimization change	1.24	0.75	1.65
Model 4	Minority status	Victimization change	1.26	0.76	1.66
	Minority status	Minority perception	2.37	0.62	3.86**
	Minority perception	Victimization change	−0.13	0.15	−0.90
	Minority status	Victimization at baseline	−5.36	1.75	−3.07**
Model 5	Minority status	Victimization at baseline	−5.40	1.77	−3.06**
	Minority status	Minority perception	2.33	0.59	3.92**
	Minority perception	Victimization at baseline	1.29	0.31	4.17**
	Minority status	Victimization change (joint predictors on the left)	1.27	0.77	1.65
	Minority perception		−0.47	0.16	−2.95**

*p < 0.05; **p < 0.01

## Results

### Self-Reported Victimization

As seen in Table 1, in the present study there was no significant association found between *Individual SES* and self-reported victimization at T1, T2, or T3. Hypothesis 1 states that *contextual minorities* experience more victimization than *contextual majorities* at baseline T1, T2 and T3, and also longitudinally over time from T1 to T2 to T3. To test this, models I and II were analyzed, separately for self-reported victimization, and for peer-reported victimization. For self-reported victimization, M1 showed (Table 2) that there was no significant intercept or slope prediction by *contextual minority* status, indicating that being a *contextual minority* in classroom as regards SES neither significantly predicts victimization experiences at T1, T2 or T3, nor predicts the change in victimization over time independently. In M2, when *contextual minority* and *minority perception* were included in the model as joint predictors, it was found that the intercept (*i*) was significantly predicted by *minority perception* but not *contextual minority* status, and the slope (*s*) was significantly predicted by *contextual minority* status but not *minority perception*, indicating that individual perceptions of minority significantly predicted baseline victimization at T1, T2, and T3, and the change in victimization behavior over time was predicted by the *contextual minority* status of an individual when corrected for *minority perception*; however, the change in victimization predicted by contextual minority status was in a negative direction, leading us to reject hypothesis 1 for self-reported victimization.

**Fig. 2:**
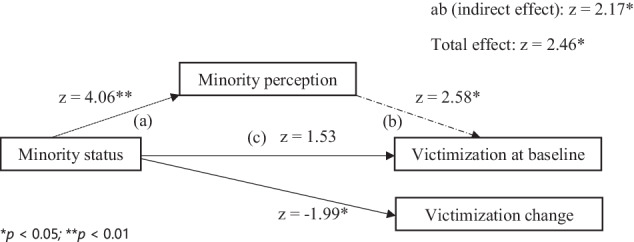
Mediation Model 3 for self-reported victimization, (**a**) direct effect from minority status to minority perception; (**b**) direct effect from minority perception to victimization; (**c**) direct effect from minority status to victimization

Hypothesis 2 of the present study states that the associations between *contextual minorities* and victimization is mediated by individuals’ *minority perceptions* at baseline and over time, such that, *contextual minorities* who perceive themselves as minorities, subsequently experience more victimization as compared to *contextual majorities*. To test this, the 4-step mediation model was examined, following Baron and Kenny’s (1986) causal effect approach. M3 (see Fig. 2) showed that for the intercept (*i*), i.e., victimization at baseline prediction, *contextual minority* status (independent variable) significantly predicted minority perception (mediator), which significantly predicted the intercept (*i)* (outcome), however, the intercept (*i*) was not directly significantly predicted by *contextual minority* status, which means that when an individual perceives themselves as minority with regard to SES, they experience more victimization at baseline. Also, there was a significant negative slope (*s*) prediction by *contextual minority* status indicating that being part of SES minority in classroom predicted less change in victimization over time. Furthermore, M3 showed that the total effect as well as the indirect effect of *contextual minority* status and *minority perception* on the intercept (*i*) was significant in the positive direction, while the direct effect of *contextual minority* and *minority perception* on the intercept was not significant, thus indicating mediation by minority perception which means that an individual experiences more victimization at baseline not only by being a part of a contextual minority but when they even perceive themselves as a minority. However, this points to change in victimization over time (*s*) such that there is a negative slope (*s*) prediction, meaning there is less change in victimization over time, by *contextual minority* status when the intercept (*i*) prediction of victimization at baseline by *contextual minority* status is fully mediated via individual’s perception.

M4 showed that *s* was neither predicted by *minority perception* nor *contextual minority*, and there was no significant total or indirect effect on *s*, thus change in victimization over time was not mediated through perception. M5 examined if slope (*s*), i.e., change in victimization over time is predicted by *contextual minority* status and *minority perception*, when the intercept (*i*), i.e., baseline victimization, is predicted by *contextual minority* status and *minority perception* with mediation through *minority perception*. M5 showed that *s* was predicted by *contextual minority* in the negative direction when corrected for *minority perception*, when the prediction of *i* by *contextual minority* was mediated by *minority perception* in the positive direction. This indicates that being a minority with regard to SES in classroom predicted less change in victimization over time over and above self-perceptions, when at baseline, being part of a minority led to perceiving self as minority and thus experiencing more victimization. M5 also showed a significant positive indirect as well as total effect of *contextual minority* on baseline victimization, and no direct effect of *contextual minority* on baseline victimization, thereby indicating that change in victimization over time was predicted by minority status when corrected for *minority perception*, and when intercept (*i*) prediction by minority status was fully mediated via individual’s perception of their minority status. Given that both M3 and M5 showed significant outcomes, a chi-square test was conducted to compare if M5 is significantly better than M3. The χ^2^ test for model difference showed that M5 did not fit significantly better than M3 (χ^2^ = 1.43, *p* > 0.05). Based on the ‘Akaike information criterion’ (AIC), M3 is the more appropriate and parsimonious model of significance for self-reported victimization, because M5 has more degrees of freedom reflecting the higher number of variables in the model. Hypothesis 2 was supported for the mediation effect of self-perceptions over time.

### Peer-Reported Victimization

A significant positive association between *Individual SES* and peer-reported victimization at T1 and T2 was found (Table 1), indicating that higher *individual SES* is significantly associated with higher victimization. Examining hypothesis 1 for peer-reported victimization, M1 showed (see Table 2) that there was a significant negative intercept prediction by *contextual minority* but no significant slope prediction, indicating that being a *contextual minority* in classroom with regards to SES significantly predicted victimization experiences at T1, T2 or T3, but did not predict the change in victimization over time. In M2, when *contextual minority* status and *minority perception* were included in the model as joint predictors, it was found that the intercept was significantly predicted by *minority perception* in the positive direction, and by *contextual minority* status in the negative direction, and the slope was significantly predicted by *minority perception* in the negative direction but not by *contextual minority* status. This indicates that individual perception of minority had a significant positive effect on baseline victimization at T1, T2, and T3, and a significant negative effect on victimization behavior over time, such that perceiving self as minority led to more victimization at baseline but less victimization over time. There was a relation between contextual minority status and victimization experiences at the intercept, but this was in a negative direction, so that hypothesis 1 was rejected for peer reported victimization.

**Fig. 3:**
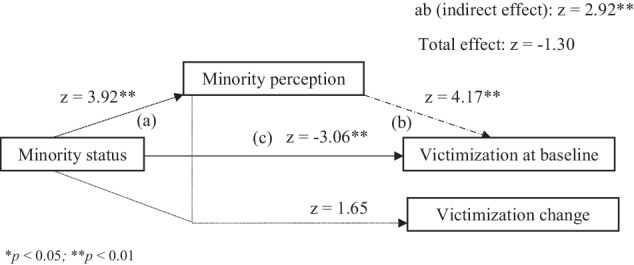
Mediation Model 5 for peer-reported victimization. **a** direct effect from minority status to minority perception; (**b**) direct effect from minority perception to victimization; (**c**) direct effect from minority status to victimization;

For the mediation model as indicated in hypothesis 2, following Baron and Kenny’s (1986) causal effect approach, M3 showed that there was significant intercept (*i*) prediction by *minority perception* in the positive direction and by *contextual minority* in the negative direction, but there was no significant slope (*s*) prediction by *contextual minority*. Furthermore, M3 showed that there was a significant positive effect of *contextual minority* on *minority perception*, and of *minority perception* on the intercept. Thus, being part of a minority with regard to SES makes the student perceive themselves as such, which leads to more victimization at baseline. However, the total effect of status and perception on the intercept was not significant, because the direct and indirect effects in this model were found to be in the opposite direction, thus, canceling each other out. M3 leads to the conclusion that individual baseline differences for victimization were significantly predicted by *contextual minority* via complete mediation through *minority perception*. M4 showed that *s* was predicted by *contextual minority* status, but not perception, and there was no significant total or indirect effect on *s*, thus change in victimization over time was not predicted by *contextual minority* status and *minority perception* through a mediation model of perception. M5 (Fig. 3) showed that slope (*s)* was predicted by *minority perception* when the prediction of intercept (*i)* by *contextual minority* was mediated by *minority perception*, and when the prediction by *contextual minority* of *s* was corrected for perception. Correcting for *minority perception* in slope prediction, M5 showed that there was a significant *s* prediction by *minority perception*, and a significant *i* prediction by *contextual minority* in the negative direction, and by *minority perception* in the positive direction. Furthermore, there was a significant positive indirect effect of *contextual minority* and *minority perception* on victimization change over time, but no significant total effect, thereby indicating that the slope was predicted by *minority perception*, when baseline victimization was predicted by *contextual minority* and *minority perception*, and this effect was fully mediated via individual’s perception of their *contextual minority* status (Fig. 3). This indicates that when adolescents who are a *contextual minority* with regard to SES, perceive themselves as such, they experience more victimization at baseline, and when this is the case, the students also experience more victimization over time. The chi-squared difference test showed that M5 is significantly better than M3 (χ^2^ = 7.71, *p* < 0.005), and thus, losing one degree of freedom to add more variables in the M5 was the more parsimonious model based on AIC in explaining variance. Hence, M5 is the better fitting model of significance for peer-reported victimization. Thus, hypothesis 2 was supported for mediating effects of self-perceptions.

## Discussion

Most research on the Social Misfit Theory was previously done in Western countries, and the theory was never before tested in India with regards to bullying victimization. In the current study it was analyzed, in a longitudinal design using both self and peer reports, whether the Social Misfit Theory could explain bullying between *contextual minorities* with regards to SES, and whether the associations between *contextual minorities* and victimization are mediated by individual *minority perception*. Overall, the results are not in line with the Social Misfit Theory. Although for the self-reports contextual minority status was related to higher victimization *at the baseline*, being a contextual minority actually predicted lower victimization *over time*. In the peer-reports, the contextual minority status was only related to lower victimization at baseline. Perceptions of self *indeed* mediated the relationship between being objective minorities and victimization experiences. However, the direction of associations were negative for direct and total effects from the predictor to the dependent variable as seen in peer-reported victimization for baseline as well as over time victimization, and for direct effects of over time victimization as seen in self-reported analyses.

### Social Misfit Theory

Overall, the results stand in contrast with the Social Misfit Theory (Wright et al., 1986) as well as with observations that a numerical minority status implies an imbalance of power, which is recognized as an antecedent of bullying victimization (Smith et al., 2018). Thus, Hypothesis 1 was not supported. A possible explanation for the reverse observation could be that being part of a minority may ward off individual tendencies of self-blame for experiencing victimization, thus, protecting the adolescent from having a reputation as a victim. Similar findings were reported with regard to ethnicity and adjustment of victims in classroom, where students who were part of an *ethnic majority* had a higher chance of adjustment problems for victims in the classroom than students belonging to an ethnic minority (Bellmore et al., 2004); Self-blaming attributions may surface more assuredly when an individual was part of the majority group holding the superior power, and was yet victimized. As opposed to this, when a victim was part of an *ethnic minority* group, experiences of victimization may have more possible attributions focusing on the context or other external characteristics rather than doubting one’s own sense of self, thereby protecting the victim’s self-esteem. In the current study it is also possible that victims adjust to their perceptions of self in the long run. It could be that victims learn to deal with bullying behaviors on their own, thus leading to lowered experiences of victimization over time (Erum, 2017), a process that could be stronger among contextual minorities.

This explanation also fits the notion that the core of the Indian mindset constitutes of discrepancies related to inconsistencies in values and belief, and contradictions in behavior (Sinha et al., 2010). There is a tendency to shape social behavior where individuals do “the needful” in an effort to accommodate to the situation. School structures in India may also be inherently contributing to such inconsistencies and contradictions. For example, schools in India can be grouped based on their funding status, where broadly, government schools are “aided” schools run by the government, while private schools are “self-aided”, or privately funded and run independently. The educational curriculum and academic syllabus of schools in India too depend on the Board of Education followed by the school, where most government schools follow the state or central government board of education syllabus while private schools follow non-governmental board of education syllabus, for example, the Cambridge board. Government aided schools typically charge lower fees to provide an opportunity to financially disadvantaged parents who seek an English-language education for their children. However, they often have limited resources, infrastructure, and facilities and due to the large population and limited resources, overcrowding could be an issue in many government schools. In contrast, elite private schools often cater to affluent families and offer top-notch facilities, infrastructure, and quality education (Thakkar, 2021). Thus, developmental outcomes may vary with respect to these structures, either due to their higher socioeconomic status or because their parents prioritize education more (Bapat, 2016).

### Perceptions of Self

In line with Hypothesis 2, perceptions of self as a minority mediated the relation between contextual minority status and victimization. This is in line with the idea that perceptions of group membership over objective group membership are an explanation for victimization (Hutnik, 2004). Both in the self- and peer reported models, perception of oneself as a minority predicted higher victimization at baseline. Paradoxically however, even though membership of a minority group predicted self-perception as a minority, which predicted higher victimization, overall being a contextual minority predicted decreased victimization over time. Potentially, a more powerful mediator not included in our design may have overshadowed the potential effects of identification as a minority. A more powerful mediator may be friendships, because children tend to form friendship with others who are similar in terms of ethnicity (Fortuin et al., 2014). These friendships form a resource for support and protection. It could be that similarly in Indian classrooms contextual SES minorities form friendship cliques. Though speculative, these friendship cliques among minorities may take time to form, which may explain why minority status predicted lower victimization over time. Thus, while hypothesis 1 stating that being a part of minority predicts more victimization experiences at baseline and over time was rejected, hypothesis 2 stating that the said direction of influence will be mediated by perceptions was supported, albeit in the reverse magnitude. Future studies not focusing only on victimization but also on friendship selection may provide more clarity.

Furthermore, the differences in model fit observed between self- and peer-reports confirm the notion that the combination of both types of reports is advised in the study of bullying victimization and its correlates (Cornell & Bandyopadhyay, 2009). In the present study, while overall patterns, especially the mediating effect of self-perceptions, did not substantially differ between the alternate reports of victimization, nuances in independent associations between self- and peer-reported victimization were observed. The use of multiple measures for bullying victimization enabled us to observe these attenuations, underlining the importance of this design characteristic of the present study. In the present study, the observations reported through peer-reports of victimization, which are typically seen as a more valid indication of bullying than self-reports (Branson & Cornell, 2009), point to the conclusion that victimization at baseline, as well as over time, is affected by perceptions of self as a minority in a classroom.

### Limitations, Conclusions, and Implications for Future Research

The present study has limitations. There was no differentiation between different forms of victimization experiences (physical, social, or relational; Malhi et al., 2015). The self-reported scale used to measure victimization experiences in the present study (Espelage & Holt, 2001), albeit a reliable and valid tool, does not operationally differentiate between victimization as being at the receiving end of bullying behaviors from being the recipients of aggression (Ostrov et al., 2019). Data on perceptions of self as a minority with regard to SES were obtained at one time-point only. However, self-perceptions have been typically found to be stable over time (Diehl et al., 2006). Furthermore, given that a past study from India suggests insignificant associations between covariates such as gender, age, and SES (outside of classroom context) (Thakkar et al., 2020b), these covariates were not examined in the present study. However, this study only focused on one study in India, so that we cannot be sure that gender, age and SES would not be covariates in other regions, or perhaps moderators leading to a decrease in victimization over time for *contextual minorities* that have not been accounted for in the present study. Additionally, explicit multilevel modeling (Enders et al., 2018) was not used to address the longitudinal and nested structure of data in the current study. However, given the negligible ICCs observed in the present study, and the use of robust standard errors with FIML estimation to correct for the nested structure of the data, the present study maintains the methodical rigor required to make unbiased inferences. Furthermore, the present study classifies individual SES into categories of low, middle, high SES to construct and examine classroom compositions and majority/minority context. However, this restricts the capacity to use SES as a continuous measure in moderator analyses, thereby limiting the ability to capture the dimensional nature of the construct (Newsom et al., 2013). We did not include gender as a moderator in our analyses, in part because our sample of girls was substantially smaller than the sample of boys. Gender may moderate processes of bullying, and more attention to gender in bullying processes has been called for (Rueger et al., 2011). It should be noted that the data used in the current study is over 10 years old. However, because studies from India about bullying are very scarce (Thakkar et al., 2020a), and SES differences are still prevalent in India, the analyses presented here still provide important information.

## Conclusion

The relation between being a contextual minority and victimization is mediated by the perception of self as a minority, so that being a contextual minority predicts higher perception as a minority, which in turn predicts higher victimization. However, surprisingly, objective status as a contextual minority over time is related to lower victimization, in both peer and self-reports. These results make it difficult to provide firm recommendations to teachers or policymakers, but they do show that minority status is related to identity in Indian youth. This fits with a large line of studies that suggest the importance of identity formation in adolescents (Vedder & Van Geel, 2017). At least teachers should be aware that context may shape identity, and in turn behavior, and respect for diversity may be a key in diminishing any negative outcomes of Identity development. Still, while self-perceptions may certainly play a role in victimization, future research is needed to further disentangle the relations between minority status and victimization.
